# Characterization of Retinal Function Using Microperimetry-Derived Metrics in Both Adults and Children With *RPGR*-Associated Retinopathy

**DOI:** 10.1016/j.ajo.2021.07.018

**Published:** 2022-02

**Authors:** Evgenia Anikina, Michalis Georgiou, James Tee, Andrew R. Webster, Richard G. Weleber, Michel Michaelides

**Affiliations:** a Moorfields Eye Hospital (E.A., M.G., J.T., A.R.B., M.M.), London, United Kingdom; b From the University College London Institute of Ophthalmology (E.A., M.G., J.T., A.R.B., M.M.), University College London, London, United Kingdom; c Department of Ophthalmology, University of Arkansas Medical Sciences, Little Rock, Arkansas, USA.; d Casey Eye Institute, Oregon Health & Science University (R.G.W.), Portland, Oregon, USA

## Abstract

**PURPOSE:**

To investigate microperimetry testing of retinitis pigmentosa GTPase regulator gene (*RPGR*)-associated retinopathy in a cohort of children and adults.

**DESIGN:**

Prospective observational case series.

**METHODS:**

The coefficient of repeatability and intraclass correlation coefficient (ICC) of mean sensitivity (MS) were calculated for mesopic microperimetry. Best-corrected visual acuity (BCVA), contrast sensitivity (CS), MS, total volume (V_TOT_), and central 3-degree field volume (V_3_) from volumetric and topographic analyses were acquired.

**RESULTS:**

The study recruited 76 individuals with *RPGR* (53 adults, 23 children). The mean follow-up period was 2.8 years. The ICC values for MS, V_TOT_, and V_3_ were 0.982 dB (95% CI, 0.969-0.989 dB), 0.970 dB-steradian (sr) (95% CI, −0.02658 to 0.03691 dB-sr), and 0.986 dB-sr (95% CI, 0.978-0.991), respectively. The *r* values for interocular MS, V_TOT_, and V_3_ were 0.97 (*P* < .01), 0.97 (*P* < .01), and 0.98 (*P* < .01), respectively, indicating strong interocular correlation. The interocular correlation of progression for MS, V_TOT_, and V_3_ was 0.81 (*P* < .01), 0.64 (*P* < .01), and 0.81 (*P* < .01), respectively. There was no statistically significant difference in the interocular progression rates for MS or V_TOT_. V_3_ did show a statistically significant difference. Most patients lost retinal sensitivity rapidly during their second and third decades of life.

**CONCLUSIONS:**

The high degree of reproducibility of results and the good interocular correlation lends this method to accurately monitoring disease progression, as well as supporting validation of the use of MP in assessing the outcomes of gene therapy clinical treatment trials.

## HIGHLIGHTS

• Retinitis pigmentosa GTPase regulator gene (RPGR)-associated retinopathy is a predominantly symmetric test.
• Microperimetry has a good test-retest reliability for the disease and can track progression of retinal functional loss.
• The high degree of reproducibility of results, good interocular correlation, and accurate tracking of change over time lends this modality well to assessing the outcomes of gene therapy trials.

X-linked retinitis pigmentosa (XLRP) is a subset of genetically heterogenous conditions that fall under the broad phenotypic group of retinitis pigmentosa (RP). Affected individuals typically present with nyctalopia and progressive peripheral visual loss. In the later stages of the condition, central vision becomes affected, resulting in severe visual impairment.^1^

XLRP accounts for 5% to 15% of all RP cases.^2^^,^^3^ Pathogenic sequence variants in the RP GTPase regulator gene (*RPGR*) have been shown to be responsible for 75% of cases of XLRP. *RPGR* variants have also been associated with cone dystrophy (COD), cone-rod dystrophy (CORD), and sector RP.^4^, ^5^, ^6^ *RPGR*-associated RP (XLRP-*RPGR*) has a particularly severe phenotype, characterized by early onset of symptoms, usually in early childhood and particularly rapid progression of visual loss. It is currently the target of several gene therapy trials (NCT04671433, NCT03252847, NCT03116113, NCT03316560, and NCT04517149) that aim to arrest progression and improve retinal function. A recent publication of the initial results of a gene therapy trial for XLRP-*RPGR* included microperimetry (MP) testing as part of the secondary outcomes.^7^ This trial used the mesopic Macular Integrity Assessment (MAIA) MP assessment (CenterVue MP Systems, Padova, Italy), which was central to achieving an objective assessment of retinal sensitivity changes after treatment and for the monitoring of inflammatory complications of gene therapy and documenting their resolution.

Retinal sensitivity measures are widely used as part of retinal functional assessment and often constitute key metrics for monitoring disease progression. Common methods for measuring retinal sensitivity include full-field dynamic and static perimetry or MP (fundus-guided perimetry). Test-retest repeatability of MP in an *RPGR* patient cohort using the MAIA system has been reported.^8^ Previously published data from our group explored repeatability, interocular symmetry, and rate of progression using full-field static perimetry in XLRP-*RPGR*.^9^ We have developed a customized testing protocol for *RPGR*-associated retinopathy using the Nidek MP1 (Nidek Technologies, Padova, Italy), aiming for a standardized and reproducible assessment of point-by-point retinal sensitivity, as surrogate measures of retinal function and disease progression. Further analysis of retinal sensitivity was undertaken using the Visual Field Monitoring and Analysis software (VFMA: Office of Technology Transfer & Business Development, Portland, OR).^10^, ^11^, ^12^ This uses all of the point-by-point retinal sensitivity measures obtained from the MP testing grid to generate a comprehensive volume plot of retinal sensitivity, which is also a more sensitive measure of change over time. A total hill of vision (V_TOT_) or any other volumetric measure of sensitivity can be generated within defined retinal areas; for example, within the central 3° (V_3_).

We aimed to characterize retinal function in detail, including disease symmetry and natural history, in a large, molecularly confirmed cohort of *RPGR*-associated retinopathy, by using a range of MP-derived metrics. The correlation of MP metrics with contrast sensitivity (CS) and best corrected visual acuity (BCVA) was investigated. This study also provides data to support the validation of MP-derived metrics as clinically meaningful end points for patient stratification and monitoring of treatment effect in both on-going and future gene therapy trials.

## METHODS

Ethical approval was received from the Moorfields Eye Hospital Ethics Committee at (London, United Kingdom) for this study. The study adhered to the Declaration of Helsinki.

### STUDY PARTICIPANTS

All participants were affected males with molecular genetic confirmation of disease-causing variants in *RPGR*. Participants aged younger than 18 years were classified as children, and those older than 18 were classified as adults.

### MOLECULAR GENETICS

All patients were recruited from the Moorfields Eye Hospital retinal genetics service. Patients were genetically screened by variable protocols, as previously described.^13^

### ASSESSMENT OF VISUAL FUNCTION

Patients attended research appointments at 6-month intervals for 2 years and consequently for annual visits. Visual function assessments included BCVA at 4 m, using the Early Treatment Diabetic Retinopathy Study (ETDRS) chart, followed by CS testing at a distance of 1 m with the Pelli-Robson chart. BCVA was recorded in logMAR units and CS as logCS units.

### ASSESSMENT OF RETINAL FUNCTION

Mesopic fundus-guided perimetry was performed using the Nidek MP-1. Pupils were dilated using phenylephrine hydrochloride 2.5% solution (Bausch & Lomb, Inc) and tropicamide 1% ophthalmic solution (Akorn, Inc). Fixation was monitored by a dedicated ophthalmic technician throughout each assessment. A single horizontal transfoveal optical coherence tomography (OCT) scan performed on a Spectralis OCT (Heidelberg Engineering) was imported into the perimeter to facilitate the accurate centration of the testing grid over the anatomical fovea. The radial pattern 44-point testing grid developed for these assessments is shown in the Supplemental Figure. Background illumination was set to 4 apostilbs (1.27 cd/m^2^) and a Goldmann size III stimulus was presented for 200 milliseconds. The testing protocol used a 4- to 2-dB full-threshold bracketing test strategy. A mean sensitivity (MS) value was automatically computed by the manufacturer's software. The intrinsic reliability reports from the device were collected; however, in the absence of robust evidence of their value, they were not used as an exclusion criterion. Fixation stability within 4° was used as a primary reliability assessment, and testing was repeated if test results fell below 90%. Those with fixation stability of less than 90% were included in the analysis only if repeat testing remained consistent in MS and the volumetric sensitivity indices. Participants with sensitivities below the testing threshold (recorded sensitivity of 0) were also excluded from further testing or analysis.

### VOLUMETRIC INDICES OF RETINAL FUNCTION

Test data were exported from the software as comma-separated value (csv) files for analysis in the VFMA software. This software generates a 3-dimensional hill-of-vision (HOV) model of the visual field and allows the calculation of a volume of sensitivity beneath the surface of the model, based upon the total sensitivity across the solid angle of the base of the test grid, in decibel-steradian (dB-sr) units.^14^ This is an objective numerical measure of the total sensitivity of the examined retinal area (termed V_total_/V_TOT_) and can be subanalyzed by area. In our study, we also analyzed the visual field contained within a central circle of 3° radius (termed V_3_), based on the total sensitivity across the solid angle of a central 6°, to reflect the function of the central visual field.

### PROGRESSION ANALYSIS

Progression rates for each individual eye were obtained from gradients of linear trend lines fitted to data points using the least-squares method. Photoreceptor degeneration and the decline of visual function in RP, both in animal models and humans, follows an exponential pattern over an extended time period.^15^, ^16^, ^17^, ^18^, ^19^, ^20^ Nevertheless, shorter follow-up periods can be successfully modeled using a linear best-fit line to capture a short-range progression snapshot.^9^ Only eyes with a minimum of 3 data points and a minimum follow-up of 1 year were included.

### DISEASE SYMMETRY

The Bland-Altman analysis was used to assess interocular differences in MS, V_TOT_, and V_3_ at baseline and for the interocular progression rates. The Spearman correlation coefficient was used to investigate interocular correlation between baseline MS, V_TOT_, and V_3_, and progression rates for V_TOT_ and V_3_.

### STATISTICAL ANALYSIS

Statistical analysis was performed with SPSS Statistics software (IBM Corp). Significance for all statistical tests was set at *P* < .05. The Shapiro-Wilk test was used to test for normality for all variables. Test-retest reliability was investigated with the intraclass correlation coefficient (ICC) based on absolute agreement and a 2-way mixed-effects model using results from the right eye to minimize the clustering effect.

## RESULTS

### DEMOGRAPHICS AND GENETICS

**FIGURE 1:**
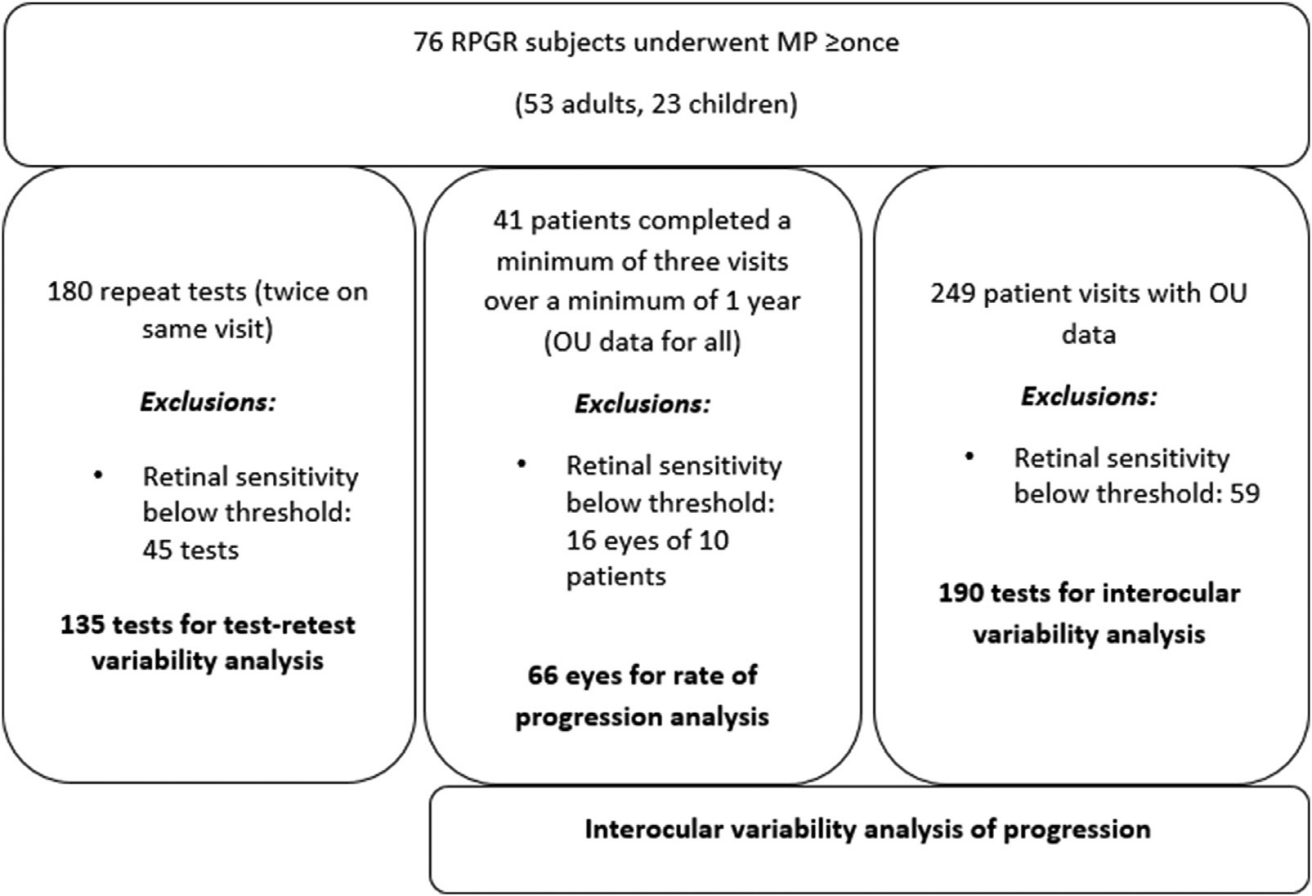
Flow chart of subject recruitment and participation in testing. MP = microperimetry; *RPGR* = retinitis pigmentosa GTPase regulator.

MP testing was performed in 76 individuals (53 adults, 23 children) with *RPGR* retinopathy. The age range of patients was 6.9 to 55.8 years (mean, 25; median, 24) at baseline testing. The mean follow-up period was 2.8 years (range, 1.2-5.3). The flow chart of patient participation is given in Figure 1.

Disease-causing variants in *RPGR* were present in the open reading frame 15 (ORF15) in 49 participants (64%) or in exons 1 to 14 in 27 (36%). Of those with variants in the ORF15 region, there were 2 patients expressing a COD phenotype, 1 patient with CORD, and 1 patient with sectoral RP. All other patients had XLRP.

### FUNCTIONAL ASSESSMENT

The baseline BCVA, CS, MS, V_TOT_, and V_3_ metrics are presented in Table 1. Annual progression rates for these parameters are also reported.

**Table 1:** Functional Assessment of Progression

			OD Progression Rate				OS Progression Rate	
Metric	OD Baseline Mean (SD)	OD Baseline Median (IQR)	Mean (SD)	Median (IQR)	OS Baseline Mean (SD)	OS Baseline Median (IQR)	Mean (SD)	Median (IQR)
BCVA, LogMAR	0.41 (0.34)	0.34 (0.34)	0.00 (0.05)	0.00 (0.04)	0.44 (0.40)	0.39 (0.28)	0.03 (0.07)	0.01 (0.07)
CS, logCS	1.19 (0.45)	1.30 (0.63)	0.02 (0.09)	0.02 (0.08)	1.15 (0.42)	1.23 (0.56)	0.00 (0.08)	0.00 (0.07)
MS, dB	6.75 (6.47)	3.9 (10.00)	0.82 (0.87)	0.74 (1.10)	6.75 (6.84)	3.30 (10.96)	0.67 (0.86)	0.54 (1.06)
V_TOT_, dB-sr	0.30 (0.36)	0.12 (0.54)	0.04 (0.06)	0.0289 (0.069)	0.31 (0.39)	0.10 (0.53)	0.04 (0.06)	0.03 (0.06)
V_3_, dB-sr	0.06 (0.06)	0.04 (0.11)	0.01 (0.01)	0.01 (0.01)	0.05 (0.06)	0.04 (0.08)	0.01 (0.01)	0.01 (0.01)

Mean and median best-corrected visual acuity (BCVA) in LogMAR units, mean sensitivity (MS) in decibels (dB) and the volumetric indices V_TOT_ and V_3_ in decibel-steradians (dB-sr) for the right eye (OD) and left eye (OS). Progression rates calculated per year. SD = standard deviation, IQR = interquartile range

### TEST-RETEST RELIABILITY

**FIGURE 2:**
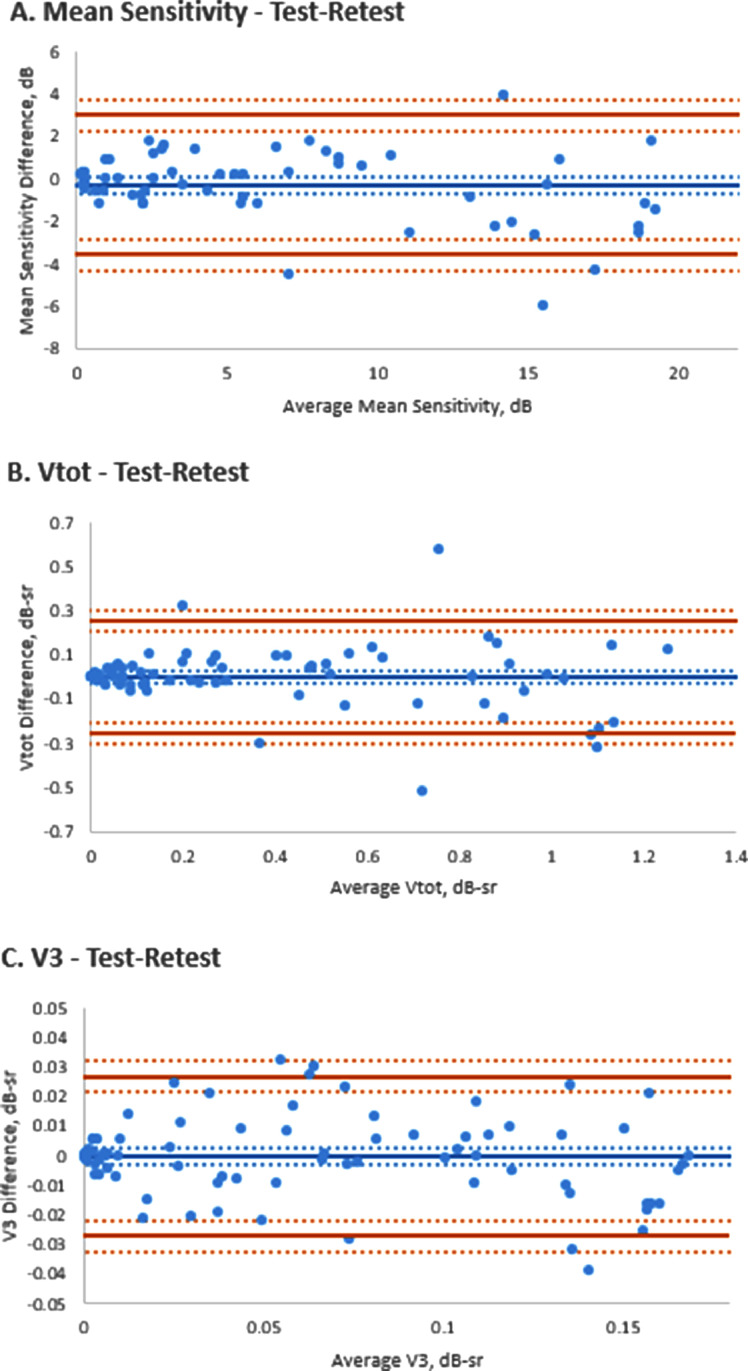
Test-retest reliability assessment. Bland-Altmann plots of the test-retest reliability of the (A) mean sensitivity measurements in dB, and the volumetric measurement in dB-steradian (dB-sr) of (B) total retinal sensitivity (V_TOT_) and (C) of the fovea-centered area of radius 3° (V_3_).

After exclusions for subthreshold sensitivity (MS value of 0 dB), 135 pairs of tests were analyzed. All paired same-day tests passing the exclusion criteria were included, not only those at baseline. The ICC values in this subgroup for MS, V_TOT_, and V_3_ were 0.982 (95% CI, 0.969-0.989), 0.970 (95% CI, −0.02658 to 0.03691), and 0.986 (95% CI, 0.978-0.991), respectively. This indicates a strong correlation between separate consecutive tests and a high test-retest reliability for this method. Bland-Altman plots for the test-retest reliability of right eye MS, V_TOT_, and V_3_ are presented in Figure 2.

### INTEROCULAR SYMMETRY

**FIGURE 3:**
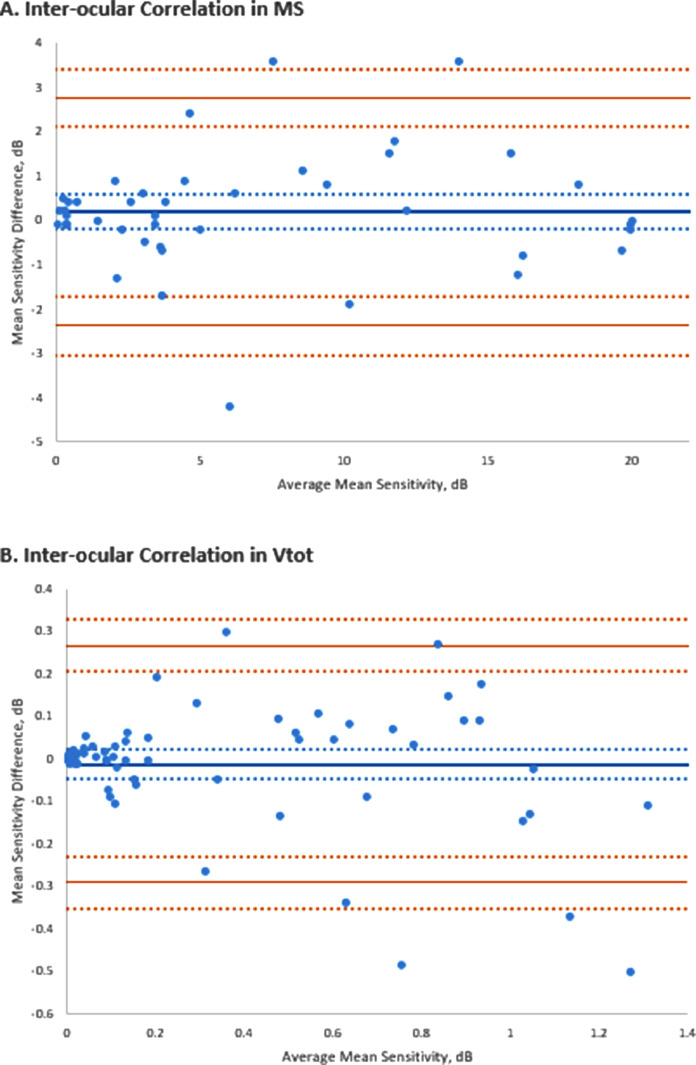
Interocular symmetry assessment. Bland-Altmann plots of the (A) interocular symmetry of the mean sensitivity (MS) and (B) the volumetric measurement of total retinal sensitivity (V_TOT_). dB-sr = in dB-steradian.

Data from 190 test pairs for right and left eyes at baseline were analyzed after the application of the aforementioned exclusion criteria. The *r* values (Pearson correlation coefficient) for interocular MS, V_TOT_, and V_3_, were 0.97 (*P* < .01), 0.97 (*P* < .01), and 0.98 (*P* < .01), respectively, indicating strong interocular correlation of all metrics. The interocular correlation (Spearman correlation coefficient) of progression for MS, V_TOT_, and V_3_ were 0.81 (*P* < .01), 0.64 (*P* < .01), and 0.81 (*P* < .01), respectively. The Bland-Altman plots for the interocular MS and V_TOT_ are presented in Figure 3. There was no statistically significant difference in the interocular progression rates for MS (*P* = .26) or V_TOT_ (*P* = .70) by paired *t* test. V_3_ did show a statistically significant difference at *P* = .01.

### RATE OF PROGRESSION

**FIGURE 4:**
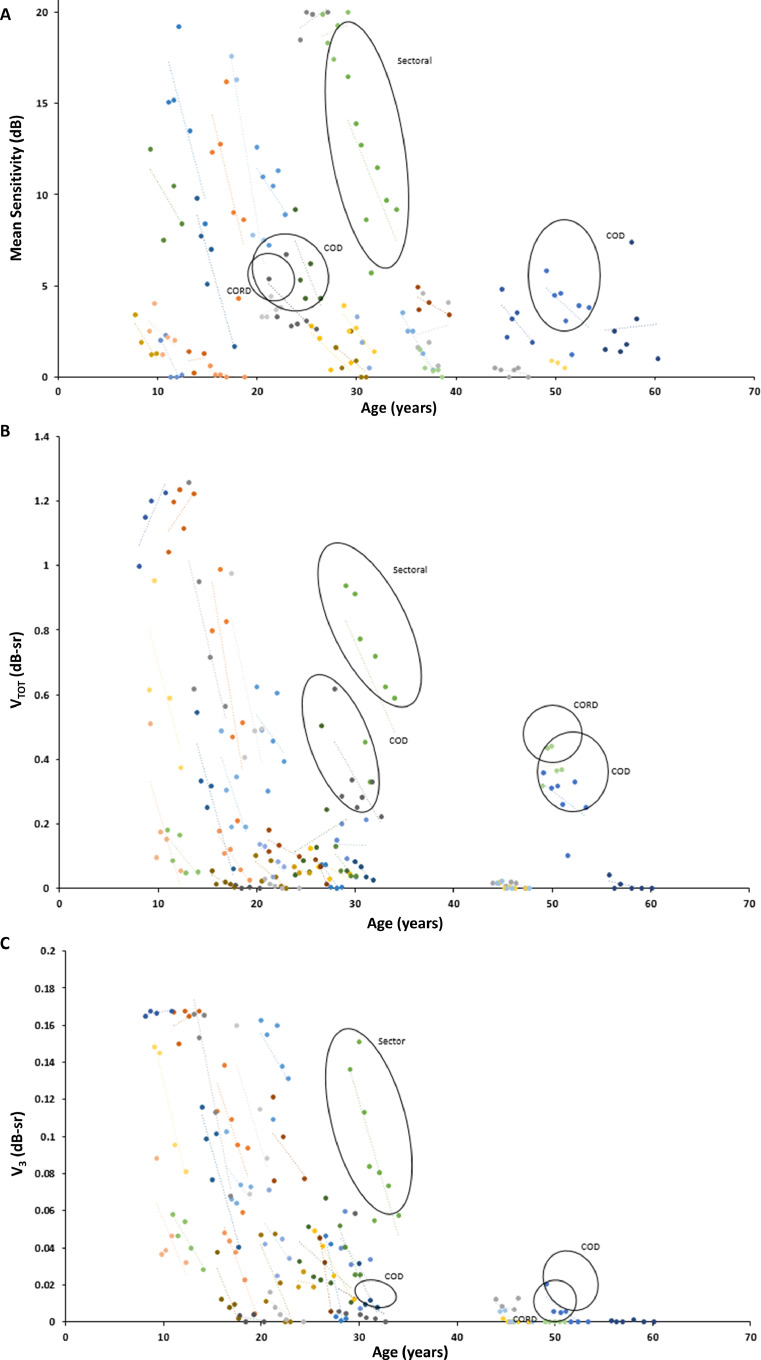
Progression of retinal functional loss. Plots with best-fit lines of all individuals (X-linked retinitis pigmentosa [XLRP], 27; cone dystrophy [COD], 2; cone-rod dystrophy [CORD], 1; and sector retinitis pigmentosa [RP], 1) in the cohort with ≥3 consecutive retinal sensitivity measurements. A. The mean sensitivity. The volumetric measurement of (B) total retinal sensitivity (V_TOT_) and (C) fovea-centered area of radius 3° (V_3_) are presented for the analyzed right eyes. dB-sr = dB-steradian.

Because there was a strong interocular correlation at baseline, only OD data were selected for analysis of progression as being representative of the cohort. The mean rates of annual progression of MS, V_TOT_, and V_3_ were 0.82 dB/y, 0.04 dB-sr/y, and 0.01 dB-sr/y respectively (Table 1). Figure 4 shows the progression plots for MS, V_TOT_, and V_3_.

The rate of progression in the ORF15 genotype subgroup was comparable to that of the subgroup with disease-causing variants in exons 1 to 14 (MS, 0.81 dB/y in both subgroups; V_TOT_, 0.042 and 0.039 dB-sr/y, respectively; and V_3_, 0.009 and 0.013 dB-sr/y, respectively). None of the differences reached statistical significance (*P=* .667 for MS, *P =* .535 for V_TOT_, and *P =* .395 for V_3_)

Most patients lost retinal sensitivity rapidly during their second and third decades of life. Patients with COD, CORD, and sectoral RP phenotypes were outliers, as would be anticipated. For example, the patient with sectoral RP demonstrated a similar characteristic rapid decline in MS, V_TOT_, and V_3_; however, this was noted later in life. One of the 2 COD patients and the CORD patient demonstrated unique relative preservation of V_TOT_ into later life, however, with characteristic reductions in MS and V_3_.

### CORRELATION WITH BCVA AND CS

The correlation between baseline BCVA and CS, and the MS, V_TOT_ and V_3_ metrics was tested using the Spearman correlation coefficient. There was a positive statistically significant correlation between baseline BCVA and CS, BCVA and MS, BCVA and V_TOT_*,* and BCVA and V_3_ (0.80, 0.62, 0.58, and 0.77, respectively). There was also a positive statistically significant correlation between baseline CS and MS, CS and V_TOT_*,* and CS and V_3_ (0.70, 0.65, and 0.85, respectively).

The correlation between progression rates of BCVA and CS, and the MS, V_TOT_, and V_3_ metrics was also investigated. There was a weak positive correlation between the annual progression rates of BCVA and CS (0.14) and weak positive correlations between the progression rates of BCVA and the MS, V_TOT_, and V_3_ metrics (0.06, 0.14, and 0.03, respectively). There was also a weak positive correlation between the annual progression rates of CS and MS, V_TOT_, and V_3_ (0.21, 0.29, and 0.26, respectively). These were not statistically significant.

## DISCUSSION

To our knowledge, this is the largest prospective report on retinal sensitivity using MP in a molecularly confirmed group of *RPGR* individuals including adults and children. Traditional assessments of visual function, such as visual acuity (VA) and CS, are not ideal for monitoring disease progression in *RPGR*, because most changes occur outside of the fovea until late in the disease course. This means that relative preservation of the VA and CS parameters does not reflect the rapid progressive changes in visual function that occurs. Furthermore, clinical treatment trials need to review the response to treatment over relatively short time periods, over which VA and CS have been observed to remain largely stable. Hence surrogate markers of retinal function need to be used for accurate monitoring.

### INTEROCULAR SYMMETRY

There was good overall symmetry in the MS and the volumetric indices of retinal function, V_TOT_, and V_3_ at baseline, as demonstrated by the *r* values of the interocular correlation coefficient.

Interocular symmetry of visual function has previously been demonstrated in individuals with *RPGR*.^9^^,^^21^ The interocular correlation of progression of the MS, V_TOT_, and V_3_ metrics was more variable. However, the variability did not reach statistical significance for MS (*P* = .264) and V_TOT_ (*P* = .705) by paired *t* test. Interocular variability in the progression of V_3_ was statistically significant at *P* = .01. What this signifies clinically is not clear; however, we postulate that the absolute values of retinal sensitivity are so small for the V_3_ metric that the effect of small differences and any outlying values is magnified and can skew the analysis.

Clinical trials of treatment frequently use fellow eyes as controls, and hence, good interocular correlation is useful in monitoring treatment response vs control progression. We believe adequate interocular symmetry is demonstrated in our participants to facilitate this; however, we would recommend acquiring sufficient baseline testing to establish a reliable and robust starting point and focusing assessments on the MS and V_TOT_ metrics. Furthermore, with high levels of interocular disease symmetry demonstrated, the advent of approved gene therapy in the future promises potentially similar benefit to both eyes from receiving treatment.

### TEST-RETEST RELIABILITY

The ICC results for MS, V_TOT_, and V_3_ indicate strong test-retest reliability. The MS is a traditional measure of visual function; however, because it is a mathematical average, it lacks the precision necessary to distinguish point-by-point sensitivity and does not possess the comprehensive reflection of total retinal sensitivity such as that achieved by the volumetric indices V_TOT_ and V_3_. Good test-retest reliability of these metrics lends support to their use in monitoring visual function progression in this cohort of patients.

MP is a faster test to perform compared with full-field perimetry such as the Octopus900 (Haag-Streit AG) and, hence, may be easier to include in testing regimens for both monitoring of progression and recording the response to treatment trials. It was also reasonably well tolerated by the children in our study, with participants as young as 8 years being able to reliably perform testing on the Nidek MP-1 in this cohort. Given the early onset of visual loss in XLRP, this lends it well to detect changes in childhood. A recent report,^8^ using the MAIA perimeter reported good test-retest reliability for testing in an XLRP-*RPGR* cohort (with no data provided on the age of these individuals, but likely to have been adults only).They found greater variability between the first 2 tests participant performs than between tests 2 and 3, indicating a learning phenomenon, resulting in the recommendation to perform 3 baseline assessments in cases of clinical trials and use the final macular sensitivity result as baseline. The ICC results for MS and the volumetric indices in our study confirm high levels of test-retest reliability for MP measurements on the Nidek MP-1 perimeter. This further supports the potential use of this device to monitor progression and treatment effect in XLRP-*RPGR*.

### RATE OF PROGRESSION

The mean rates of annual progression of MS, V_TOT_, and V_3_ were 0.82 dB/y, 0.04 dB-sr/y, and 0.01 dB-sr/y, respectively, in our cohort. This is comparable to the progression rates previously reported by Tee and associates,^8^ based on full-field static perimetry using the Octopus 900 perimeter, with the V_30_ and V_5_ mean annual rates of progression of right eyes being reported as 0.6819 and 0.0056 dB-sr, respectively. Together, these findings support the anticipated progression of the natural history of XLRP-*RPGR* and lend weight to prognostic information that can be imparted to patients.

The correlation of the baseline BCVA and CS with the MS and the volumetric indices is robust and statistically significant. The greatest correlation was observed between baseline BCVA and CS, with the second strongest correlation being noted between both BCVA and V_3_*,* and CS and V_3_. This implies that baseline BCVA, CS, and V_3_ may be useful as surrogate markers for disease severity at baseline. However, there was only a weak, not statistically significant correlation, between the progression rates of BCVA and CS and the MS and V_TOT_. This supports the observation that progression rates of central visual loss can differ significantly from full visual field loss in this primarily rod-centric phenotype, with central macular function remaining relatively preserved until the latest stages of disease.

It is notable that the progression rates of loss of retinal sensitivity are comparable between the molecularly heterogenous cohort of XLRP patients, with similar progression rates being recorded between the ORF15 and the exons 1 to 14 subgroups. A previous study investigating progression rates using full-field Octopus perimetry found an increased rate of progression (although not statistically significant) in the exon 1 to 14 subgroup, although their findings may have been confounded by an age disparity between the 2 molecularly distinct cohorts.^8^ Another study, carried out in the Asian population, also supported the finding that patients with variants in exons 1 to 14 retained less visual acuity than patients with ORF15 variants and deteriorated faster.^22^ Similar findings were identified in a cohort of 14 Japanese patients.^23^

### LIMITATIONS

The study has several limitations. For evaluating progression rates, a longer time period would be desirable, which would allow modeling of progression according to established exponential models. In our study with shorter follow-up, a linear trend line fit was more appropriate for estimating progression rates. The Nidek-MP-1 has a relatively narrow dynamic range of 0 to 20 dB, and this meant that younger participants in our cohort were sometimes able to reach the ceiling of sensitivity, and maximum sensitivity values were recorded for sequential tests. Similarly, older patients frequently fell below the sensitivity threshold, and values of 0 were generated for their retinal sensitivity results, despite some preserved level of retinal function (BCVA and CS). These floor and ceiling effects could be addressed with wider bracketing strategies for the testing protocol, which is not possible to achieve on the Nidek MP-1. Newer MP devices have a wider dynamic range of stimuli intensities. It is nevertheless useful to analyze the data acquired on the Nidek MP-1, because most of our cohort of patients fell well within its sensitivity brackets, and so meaningful progression data were collected.

Furthermore, the testing protocol used only a mesopic testing strategy. The latest MP devices can perform across a range of illuminations and record sensitivities under photopic, mesopic, and dark-adapted scotopic conditions, as well as using rod- and cone-specific color stimuli (cyan and red, respectively). XLRP-*RPGR* is a condition affecting first the rod and subsequently cone system. It is therefore of interest to specifically probe the rod system in XLRP-*RPGR* to more fully assess disease progression, particularly in the early stages, and directly probe the cone system for later disease stages.

The Nidek-MP-1 tests a mixture of rod and cone function under standard mesopic protocol conditions. An alternative to the latest MP devices may be the dark-adapted chromatic Medmont M700 perimeter (Medmont International Pty Ltd), which has been designed to probe different photoreceptor mechanisms^24^^,^^25^ but requires further investigation. Further exploration of retinal function in affected females with *RPGR* retinopathy will be of value^26^ as well as correlation of MP with advanced cellular imaging techniques.^27^^,^^28^

In conclusion, we report detailed findings on interocular symmetry, test-retest reliability, and progression of retinal functional loss, using mesopic MP in the largest molecularly confirmed *RPGR* cohort to date, including both adults and children. The high degree of reproducibility of results, good interocular correlation, and accurate tracking of change over time lends this method well to monitoring disease progression as well as supporting the validation of the use of MP in assessing the outcomes of gene therapy trials.

## Appendix Supplementary materials

Image, image 1
