# Supplementary material for: Characterization of Retinal Function Using Microperimetry-Derived Metrics in Both Adults and Children With RPGR-Associated Retinopathy
Source: Am J Ophthalmol. 2022 Feb;234:81–90. doi: 10.1016/j.ajo.2021.07.018 (PMC8847997; doi:10.1016/j.ajo.2021.07.018)
Supplement: Supplementary file 1 [file mmc1.pdf]

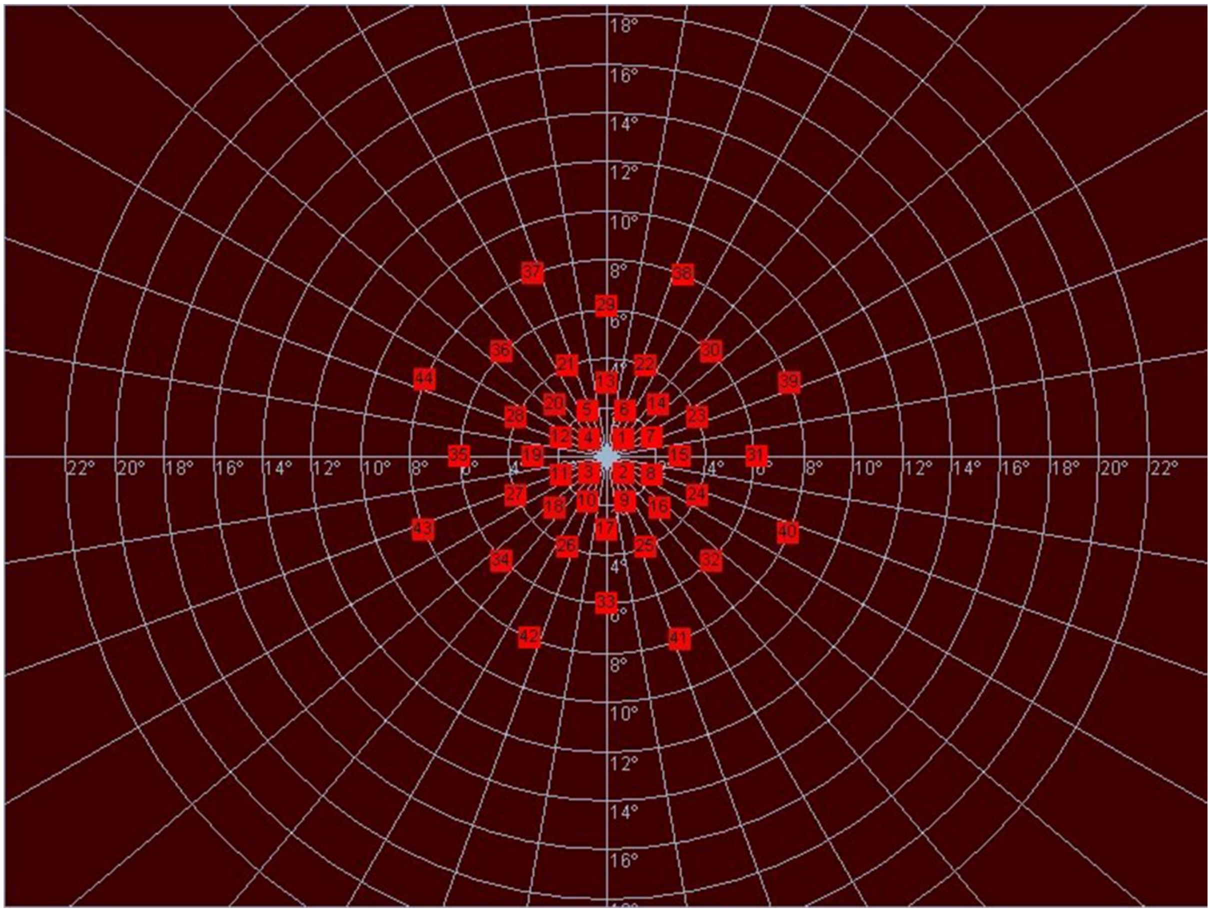

Supplementary Figure 1: Microperimetry testing grid pattern.

The grid consisted of 44 test locations, and was centrally condensed with radial organization to cover the fovea and peri-foveal region.
